# Exogenous melatonin promotes growth and sucrose metabolism of grape seedlings

**DOI:** 10.1371/journal.pone.0232033

**Published:** 2020-04-23

**Authors:** Lisha Zhong, Lijin Lin, Liu Yang, Ming’an Liao, Xun Wang, Jin Wang, Xiulan Lv, Honghong Deng, Dong Liang, Hui Xia, Yi Tang

**Affiliations:** 1 College of Horticulture, Sichuan Agricultural University, Chengdu, Sichuan, China; 2 Institute of Pomology and Olericulture, Sichuan Agricultural University, Chengdu, Sichuan, China; Huazhong Agriculture University, CHINA

## Abstract

Melatonin (MT) has many important functions in plants. In this study, different concentrations of MT (0, 50, 100, 150, and 200 μmol/L) were sprayed on grape seedlings, and its effects on plant growth and sucrose metabolism were determined. The results show that there was a mutual influence and promotional relationship between growth and sugar metabolism in grape seedlings. The MT treatments promoted the development and growth of grape seedlings by increasing their biomass and promoting the photosynthetic performance of leaves. This resulted in increased nutrient absorption and a greater ability to compete for resources. The increase in photosynthesis resulted in greater sucrose production. The MT treatments increased the activities of enzymes related to sucrose metabolism, so that a large amount of sucrose was hydrolysed into glucose and fructose to meet the rapid growth requirements of grape seedlings. The increased total soluble sugars contents and increased activities of antioxidant enzymes resulted in greater resistance of grape seedlings, and greater adaptability to environmental changes. In general, MT treatments had beneficial effects on grape seedling growth, glucose metabolism, and resistance. Under these conditions, foliar spraying with MT at 150 μmol/L had the best effects.

## Introduction

Melatonin (N-*acetyl*-5-methoxytryptamine, MT) was first found in the bovine pineal gland, and was once thought to be an endogenous hormone restricted to animals [1]. Melatonin was first detected in plants in 1995 [2], and since then, it has been widely found in the roots, leaves, fruits, and seeds of many higher plants [3]. It has many important functions in plants, ranging from the promotion of seed germination to the control of senescence [4–5]. Studies have shown that MT can regulate plant growth and development. Its characteristics appear to be similar to those of the auxin, indole-3-acetic acid (IAA). At low concentrations, MT promotes plant growth, while at high concentrations it has the opposite effect [6–7]. Melatonin also can regulate flowering [8] and the development of reproductive organs in plants [9]. It can increase photosynthetic efficiency and delay leaf senescence so that the leaves maintain strong photosynthesis for longer [10–11]. It can delay senescence in peach fruit by maintaining cell membrane integrity [12]. Another important function of MT in plants is to act as the first line of defence against oxidative stress in the internal and the external environment [13]. The antioxidant capacity of MT is related to its direct scavenging of free radicals, and its ability to activate antioxidant enzymes and protect those enzymes against oxidative damage, increase the efficiency of the mitochondrial electron transport chain, and reduce electron leakage [14–15].

Sugar metabolism is an important process in plant growth and development. In most higher plants, sucrose is the main form of photosynthetic product used for long-distance transport in the phloem, and for carbohydrate storage [16]. Plants use CO_2_ to synthesise carbohydrates in plant leaves, sheaths, and green stems (“source” tissues) during photosynthesis. After long-distance transport, carbohydrates are consumed or stored in “sink” tissues such as developing tissues, pollen, and fruit. The transport of sucrose from the “source” to “sink” includes three steps: phloem loading in the “source” tissues; transport via the vascular bundles; and phloem unloading in the “sink” tissues [17]. During these processes, the metabolism and accumulation of sucrose is controlled by different enzymes including invertase, sucrose synthase (SS), and sucrose phosphate synthase (SPS). Invertases hydrolyse sucrose into glucose and fructose [18], and can be classified as acid invertase (AI) and neutral invertase (NI) according to the pH for optimum activity. Sucrose synthase catalyses a reversible reaction; it catalyses both the synthesis of sucrose and the hydrolysis of sucrose [19]. The enzyme mainly responsible for sucrose synthesis is SPS. To date, most research on the effects of MT on fruit trees have focused on the fruit. For example, studies have shown that MT can promote fruit ripening [20] and extended the post-harvest shelf-life of fruit [21]. However, no previous studies have focused on the effects of MT on sugar metabolism in fruit tree seedlings. Sugar metabolism and plant growth are inseparable. Grape is one of the main cultivated fruits in China, and although the area of grape cultivation has increased, the quality of seedlings is irregular. Therefore, it is necessary to explore ways to improve the quality of grape seedlings. In this experiment, we determined the effects of MT treatments of grape seedlings on their growth and sucrose metabolism. The overall aim of our research was to provide a reference for the production of high-quality grape seedlings.

## Materials and methods

### Ethics statement

The grape variety was “Xiahei”, which was obtained from the vineyard of the Modern Agricultural Research and Development Base of Sichuan Agricultural University. This site belongs to Sichuan Agricultural University, which is authorized by the government of Chengdu City. The site is not privately owned or protected. No specific permits were required for the described field studies. During the experiment, no other specific permissions were required because we conducted normal agricultural activities and no endangered or protected species were involved.

### Materials

The grape variety was “Xiahei”, which was obtained from the vineyard of the Modern Agricultural Research and Development Base of Sichuan Agricultural University, Chongzhou, Sichuan Province. The grape seedlings used in the experiment were annual cuttings. During the winter pruning of grapes, annual grape vines with healthy, strong, and consistent growth were collected and stored in wet sand. In March 2019, the grape vines were taken out, cut into shoots approximately 15 cm in length, and the shoots were inserted into the seedling substrate (nutrient soil: vermiculite = 1:1). The seedlings were cultivated in a greenhouse under the following conditions: 14-h days at 25°C, relative humidity 70%, 4000 Lux; and 10-h nights at 20°C, relative humidity 90%, 0 Lux [22].

The MT used in the experiment was purchased from the Beijing Solarbio Science and Technology Co., Ltd. (Beijing, China). The soil used in the experiment was sandy soil with the following basic chemical properties: pH 7.71, organic matter 15.29 g/kg, total nitrogen 1.850 g/kg, total phosphorus 11.88 g/kg, total potassium 15.38 g/kg, alkali soluble nitrogen 87.99 mg/kg, available phosphorus 55.78 mg/kg, available potassium 41.96 mg/kg, water-soluble calcium 0.213 mg/g, water-soluble magnesium 0.028 mg/g, water-soluble potassium 0.016 mg/g, and water-soluble sodium 0.029 mg/g. The basic soil properties were determined according to Zhang (2011) [23].

### Experimental design

The experiment was conducted in the light culture room of the Fifth Teaching Building at the Chengdu Campus of Sichuan Agricultural University from March to August 2019. In March 2019, the soil was crushed and mixed, and then 3.0 kg soil was placed into 210 model plastic pots (11-cm tall, 18-cm diameter), then watered until the soil was completely moist. Healthy, consistent grape cutting seedlings with 15-cm long new shoots were selected, washed to remove the substrate from roots, and then transplanted into the prepared soil. Each pot contained three grape seedlings. The grape seedlings were cultivated in the light culture room under the following conditions: 14-h day (25°C), 10-h night (20°C), with luminance of 4000 lx. After the grape seedlings had become established, different concentrations of MT solution were sprayed onto whole grape seedlings (including onto both sides of the leaves, and on the stems). The concentrations of MT tested in this study were 0, 50, 100, 150 and 200 μmol/L, with water as the control. Each treatment had three replicates. From April 8, 2019, the seedlings were sprayed once every 7 d, each time with 25 ml MT solution per treatment. The seedlings were sprayed a total of four times. When spraying the MT solution, the different concentrations of MT were separated to avoid affecting the experimental results. The grape seedlings were cultivated according to standard management procedures. The positions of the pots were completely random, and pots were spaced 15 cm apart. The pots were moved every 7 d, and watered as required. Weeds, pests, and diseases were controlled.

### Sample analysis

After 60 d, samples were collected to measure various parameters, as listed below.

1. Photosynthetic parameters were measured using an LI-6400 portable photosynthesis system (LI-COR, USA), and included the net photosynthetic rate (Pn), transpiration rate (Tr), stomatal conductance (Gs), CO_2_ concentration of intercellular (Ci), and vapor pressure deficit of leaf (Vpdl) of the functional leaves of grape seedlings.
2. Mature leaves (0.1 g) of grape seedlings were collected for the determination of photosynthetic pigments (chlorophyll *a*, chlorophyll *b*, and carotenoid) contents [24].
3. The upper young parts of leaves (0.2 g) were collected to determine the activities of superoxide dismutase (SOD), peroxidase (POD) and catalase (CAT), and the contents of the soluble protein and malondialdehyde (MDA) [24].
4. The mature leaves (0.1 g) of grape seedlings were collected for the determination of electrical conductivity [24].
5. Mature leaves (1 g), stems (1 g) and roots (1 g) were separately collected to determine the activities of AI, NI, SS, and SPS in grape seedlings. The same crude enzyme solution was used to measure AI and NI activities, and a different crude enzyme solution was used to measure sucrose synthase in cleavage direction (SSc), sucrose synthase in synthetic direction (SSs), and SPS activities. The extraction of invertase was carried out according to Kubo et al. (2001) [25] and Zhao et al. (2001) [26], and AI and NI activities were determined according to Zhao et al. (2001) [26]. The extraction of SPS and SS was carried out according to Zhao et al. (2001) [26] and Gong et al. (2004) [27], and SSc, SSs, and SPS activities were determined according to Zhao et al. (2001) [26] and Lowell et al. (1989) [28]. Because the enzymes are easily inactivated and denatured, the entire experimental procedure was completed at 0–4°C. Afterwards, the whole plants were harvested. The roots, stems, and leaves of grape seedlings were separated and washed with tap water and then with deionised water. The samples were dried at 110°C for 15 min and then dried to a constant weight at 75°C.

The dry samples were further analysed to determine the following indicators:

6 The biomass of roots, stems and leaves.
7 Sugar content: The dried tissue samples were ground and passed through a 0.149-mm mesh nylon sieve. Then, 0.1 g dry samples of roots, stems, and leaves were used to determine the contents of total soluble sugars, fructose, sucrose, and reducing sugars [29].

### Statistical analyses

Statistical analyses were performed using SPSS 22.0 statistical software (IBM Inc., Chicago, IL, USA). Data were analysed using a one-way analysis of variance with the least significant difference test (*p* ≤ 0.05). The root/shoot ratio, total chlorophyll content, leaf relative conductivity, and the glucose content were calculated as follows: root/shoot ratio = root biomass/(stem + leaf) biomass [30–32], total chlorophyll content = chlorophyll *a* content *+* chlorophyll *b* content, leaf relative conductivity = conductivity before boiling water bath/conductivity after boiling water bath×100% [24], and glucose content = reducing sugar content-fructose content [33].

## Results

### Biomass of grape seedlings

**Fig 1:**
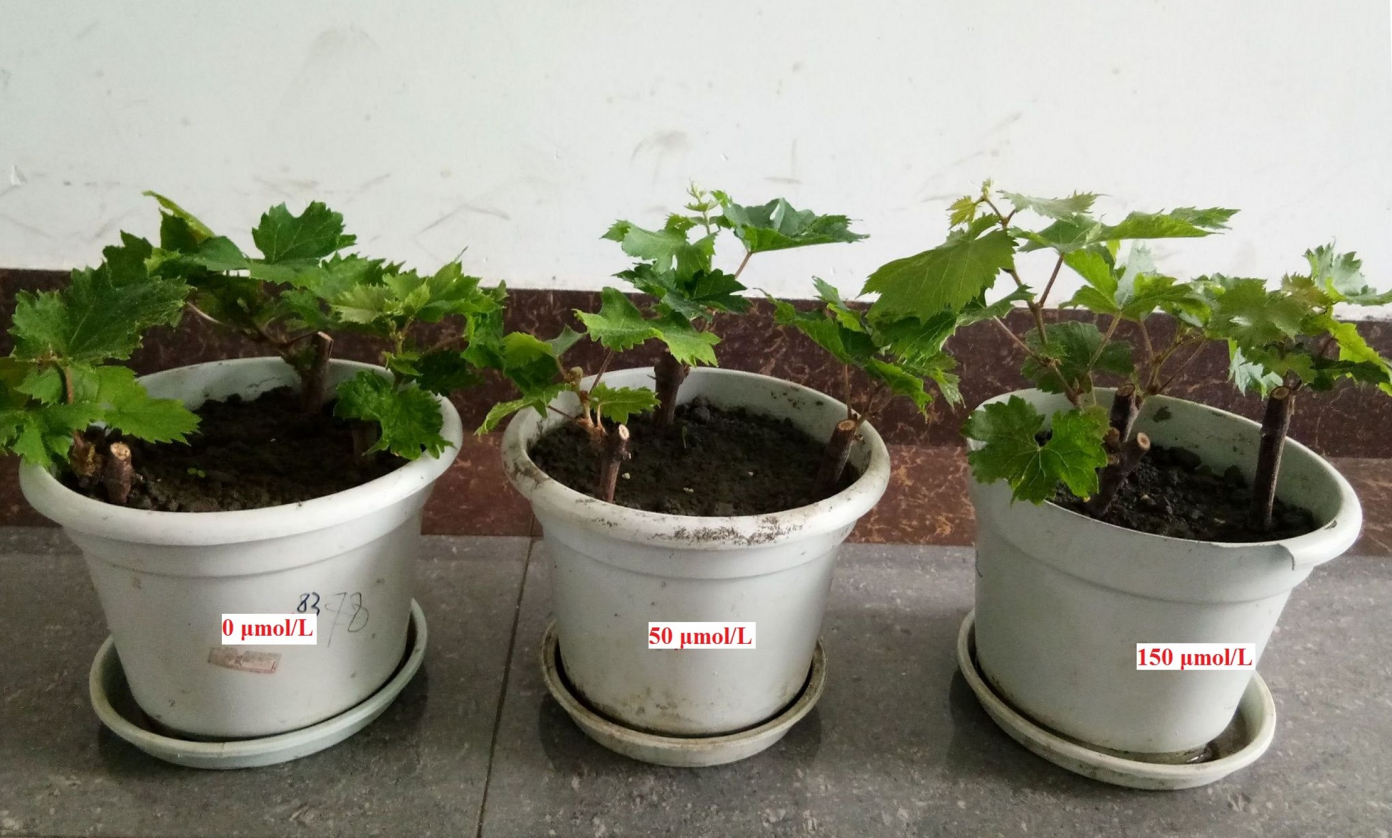
Effects of MT on the growth of grape seedlings.

The biomasses of the roots, stems, leaves, and whole plants were significantly higher in the MT treatments than in the control (*p* < 0.05, Table 1, Fig 1). At MT concentrations of ≤ 150 μmol/L, the biomass of roots, stems, leaves, and whole plants increased with increasing MT concentrations, peaking in the 150 μmol/L MT treatment. In the 150 μmol/L MT treatment, the biomasses of roots, stems, leaves, and whole plants of the grape seedlings were increased by 54.15% (*p* < 0.05), 77.78% (*p* < 0.05), 28.41% (*p* < 0.05), and 40.03% (*p* < 0.05), respectively, compared with the control. The significant increase in the biomass of the grape seedlings showed that MT promoted the growth of grape seedlings. In addition, the root/shoot ratio of grape seedlings was higher in the MT treatments than in the control, indicating that MT treatments promoted root growth more than shoot growth.

**Table 1:** Biomass of grape seedlings.

MT concentration (μmol/L)	Roots (g/plant)	Stems (g/plant)	Leaves (g/plant)	Whole plants (g/plant)	Root/shoot ratio
0	0.530±0.015c	0.099±0.002c	0.954±0.012c	1.584±0.014d	0.504
50	0.706±0.021b	0.142±0.003b	1.091±0.030b	1.938±0.047c	0.572
100	0.735±0.026b	0.170±0.006a	1.172±0.036a	2.077±0.064b	0.547
150	0.817±0.025a	0.176±0.007a	1.225±0.020a	2.218±0.047a	0.583
200	0.733±0.019b	0.142±0.005b	1.178±0.044a	2.053±0.036b	0.556

Values are means (±SE) of three replicate pots. Different lowercase letters within a column indicate significant differences based on one-way analysis of variance in SPSS 22.0 followed by the least significant differences (*p* ≤ 0.05).

### Photosynthetic parameters of grape seedlings

The photosynthetic parameters of grape seedling leaves were significantly higher in the MT treatments than in the control (Table 2). As the MT concentration increased, the net photosynthesis (Pn) of grape seedling leaves increased and then decreased after reaching a peak in the 150 μmol/L MT treatment. The Pn of grape seedling leaves was significantly higher in all the MT treatments than in the control (*p* < 0.05). The stomatal conductance (Gs) of the leaves fluctuated as the MT concentration increased. The Gs of the grape seedling leaves was significantly higher in all the MT treatments, except for the 100 μmol/L treatment, than in the control. The highest Gs of grape seedling leaves was in the 150 μmol/L MT treatment. The trends of intercellular CO_2_ concentration (Ci) and transpiration rate (Tr) in grape seedling leaves were similar, first increasing as the MT concentration increased, then increasing more slowly with higher concentrations, and finally decreasing in the 200 μmol/L MT treatment. Except for the 50 μmol/L MT treatment, all other MT treatments increased the Ci of grape seedling leaves, compared with the control. The Tr of grape seedling leaves was higher in all the MT treatments than in the control. Overall, the highest Pn, Gs, Ci, and Tr were in the 150 μmol/L MT treatment, and were increased by 81.18% (*p* < 0.05), 15.46% (*p* < 0.05), 9.79% (*p* < 0.05), and 14.98% (*p* < 0.05), respectively, compared with the control. The vapor pressure deficit (Vpdl) of grape seedling leaves showed an opposite trend to that of Pn: it tended to decrease with increasing MT concentrations, then increased after its lowest value in the 150 μmol/L treatment when it was 17.06% (*p* < 0.05) lower than that in the control.

**Table 2:** Photosynthetic parameters of grape seedlings.

MT concentration (μmol/L)	Pn (μmol CO_2_/m^2^/s)	Gs (mol H_2_O/m^2^/s)	Ci (μmol CO_2_/mol)	Tr (mmol H_2_O/m^2^/s)	Vpdl (kPa)
0	3.194±0.081d	0.207±0.006c	343.1±8.164d	2.378±0.076d	1.272±0.024a
50	4.629±0.109c	0.226±0.008ab	352.2±8.387cd	2.598±0.077c	1.261±0.025a
100	5.349±0.095b	0.216±0.006bc	369.1±6.160ab	2.782±0.069ab	1.154±0.026b
150	5.787±0.074a	0.239±0.010a	376.7±7.844a	2.884±0.091a	1.055±0.023c
200	5.324±0.104b	0.229±0.008ab	358.3±7.582bc	2.649±0.075bc	1.251±0.028a

Values are means (±SE) of three replicate pots. Different lowercase letters within a column indicate significant differences based on one-way analysis of variance in SPSS 22.0 followed by the least significant differences (*p* ≤ 0.05). Pn = net photosynthetic rate, Tr = transpiration rate, Gs = stomatal conductance, Ci = CO_2_ concentration of intercellular, and Vpdl = vapor pressure deficit of leaf.

### Photosynthetic pigment contents in grape seedlings

The contents of chlorophyll *a*, chlorophyll *b*, and total chlorophyll in grape seedlings leaves were significantly increased in the MT treatments, compared with the control. The contents of all photosynthetic pigments tended to increase as the MT concentration increased, and then decreased after reaching peaks (Table 3). The chlorophyll *a* content peaked in the 150 μmol/L treatment, and was lower in the 200 μmol/L MT treatment. The chlorophyll *b* content peaked in the 100 μmol/L MT treatment, but was not significantly different from that in the 150 μmol/L MT treatment, and was significantly lower in the 200 μmol/L MT treatment. The treatments were ranked, from highest total chlorophyll content to lowest, as follows: 150 μmol/L > 100 μmol/L ≈ 200 μmol/L ≈ 50 μmol/L > 0 μmol/L. The highest concentrations of chlorophyll *a*, chlorophyll *b*, and total chlorophyll in the leaves were in the 150 μmol/L treatment, and were increased by 25.73% (*p* < 0.05), 29.60% (*p* < 0.05), and 26.67% (*p* < 0.05), respectively, compared with the control. The chlorophyll *a*/*b* ratio of grape seedlings leaves was lower in the MT treatments than in the control, because the increase in chlorophyll *b* content was greater than the increase in chlorophyll *a* content. The trend in carotenoid content in leaves under different MT treatments was similar to that of chlorophyll *b* content. The highest carotenoid contents in leaves were in the 100 and 150 μmol/L treatments, and were increased by 14.29% (*p* < 0.05) and 18.01% (*p* < 0.05), respectively, compared with the control. Those values were not significantly different from each other.

**Table 3:** Photosynthetic pigment content in grape seedling leaves.

MT concentration (μmol/L)	Chlorophyll *a* (mg/g)	Chlorophyll *b* (mg/g)	Total chlorophyll (mg/g)	Chlorophyll *a*/*b* ratio	Carotenoids (mg/g)
0	0.688±0.019d	0.223±0.004c	0.911±0.023d	3.086	0.161±0.006d
50	0.749±0.024c	0.264±0.003b	1.013±0.026c	2.840	0.178±0.007bc
100	0.817±0.016b	0.282±0.005a	1.099±0.021b	2.892	0.184±0.005ab
150	0.865±0.030a	0.289±0.008a	1.154±0.038a	2.999	0.190±0.006a
200	0.785±0.027bc	0.262±0.008b	1.047±0.034bc	2.991	0.171±0.004cd

Values are means (±SE) of three replicate pots. Different lowercase letters within a column indicate significant differences based on one-way analysis of variance in SPSS 22.0 followed by the least significant differences (*p* ≤ 0.05).

### Antioxidant enzyme activities of grape seedlings

The activities of antioxidant enzymes (SOD, POD, and CAT) in grape seedling leaves were significantly higher in the MT treatments than in the control (*p* < 0.05, Table 4). The activities of SOD and POD increased as the MT concentration increased, reaching maximum activities in the 150 μmol/L treatment, and then decreased. The activities of SOD and POD in the 150 μmol/L MT treatment were increased by 73.36% (*p* < 0.05) and 91.32% (*p* < 0.05), respectively, compared with the control. The CAT activity increased as the MT concentration increased, with peak activity in the 100 μmol/L MT treatment. There was no significant difference in CAT activity between the 100 μmol/L and 150 μmol/L MT treatments, but it was significantly lower in the 200 μmol/L MT treatment. In the 100 and 150 μmol/L MT treatments, the CAT activities were increased by 42.98% (*p* < 0.05) and 45.42% (*p* < 0.05) respectively, compared with the control.

**Table 4:** Antioxidant enzyme activity in grape seedling leaves.

MT concentration (μmol/L)	SOD activity (U/g)	POD activity (U/g/min)	CAT activity (mg/g/min)
0	140.4±4.713e	1625±35.14d	0.698±0.017d
50	153.1±5.101d	2423±71.54c	0.752±0.023c
100	232.6±2.751b	2620±44.39b	0.998±0.026a
150	243.4±7.061a	3109±62.17a	1.015±0.034a
200	193.1±4.136c	2681±69.48b	0.857±0.028b

Values are means (±SE) of three replicate pots. Different lowercase letters within a column indicate significant differences based on one-way analysis of variance in SPSS 22.0 followed by the least significant differences (*p* ≤ 0.05). SOD = superoxide dismutase, POD = peroxidase, and CAT = catalase.

### Osmotic adjustment substance content and relative conductivity of grape seedlings

The soluble protein contents of grape seedling leaves were significantly higher in all the MT treatments than in the control, and the MDA contents and the relative conductivity were generally lower (Table 5). The soluble protein contents were highest in the 100 μmol/L and 150 μmol/L MT treatments, and were increased by 30.13% (*p* < 0.05) and 30.61% (*p* < 0.05), respectively, compared with the control. Those values were not significantly different from each other. The MDA content in the leaves was higher in the control than in all the MT treatments except for the 50 μmol/L MT treatment. The lowest MDA content in the leaves was in the 150 μmol/L MT treatment, and was 28.80% (*p* < 0.05) lower than that in the control (*p* < 0.05). The differences in the relative conductivity of grape seedlings leaves were not significantly different among the MT treatments, or between the control and all the MT treatments except for the 150 μmol/L MT treatment. In the 150 μmol/L MT treatment, the relative conductivity of the leaves was 4.75% (*p* < 0.05) lower than that of the control.

**Table 5:** Osmotic adjustment substance content and relative conductivity of grape seedling leaves.

MT concentration (μmol/L)	Soluble protein content (mg/g)	MDA content (μmol/kg)	Relative conductivity (%)
0	5.825±0.111d	33.44±1.013a	96.66±2.533a
50	6.528±0.197c	32.12±0.797a	93.21±2.316ab
100	7.580±0.224a	27.18±0.763c	92.73±2.098ab
150	7.608±0.168a	23.81±0.333d	92.07±2.113b
200	7.025±0.193b	30.11±0.846b	92.93±2.438ab

Values are means (±SE) of three replicate pots. Different lowercase letters within a column indicate significant differences based on one-way analysis of variance in SPSS 22.0 followed by the least significant differences (*p* ≤ 0.05). MDA = malondialdehyde.

### Sugar contents in grape seedlings

**Fig 2:**
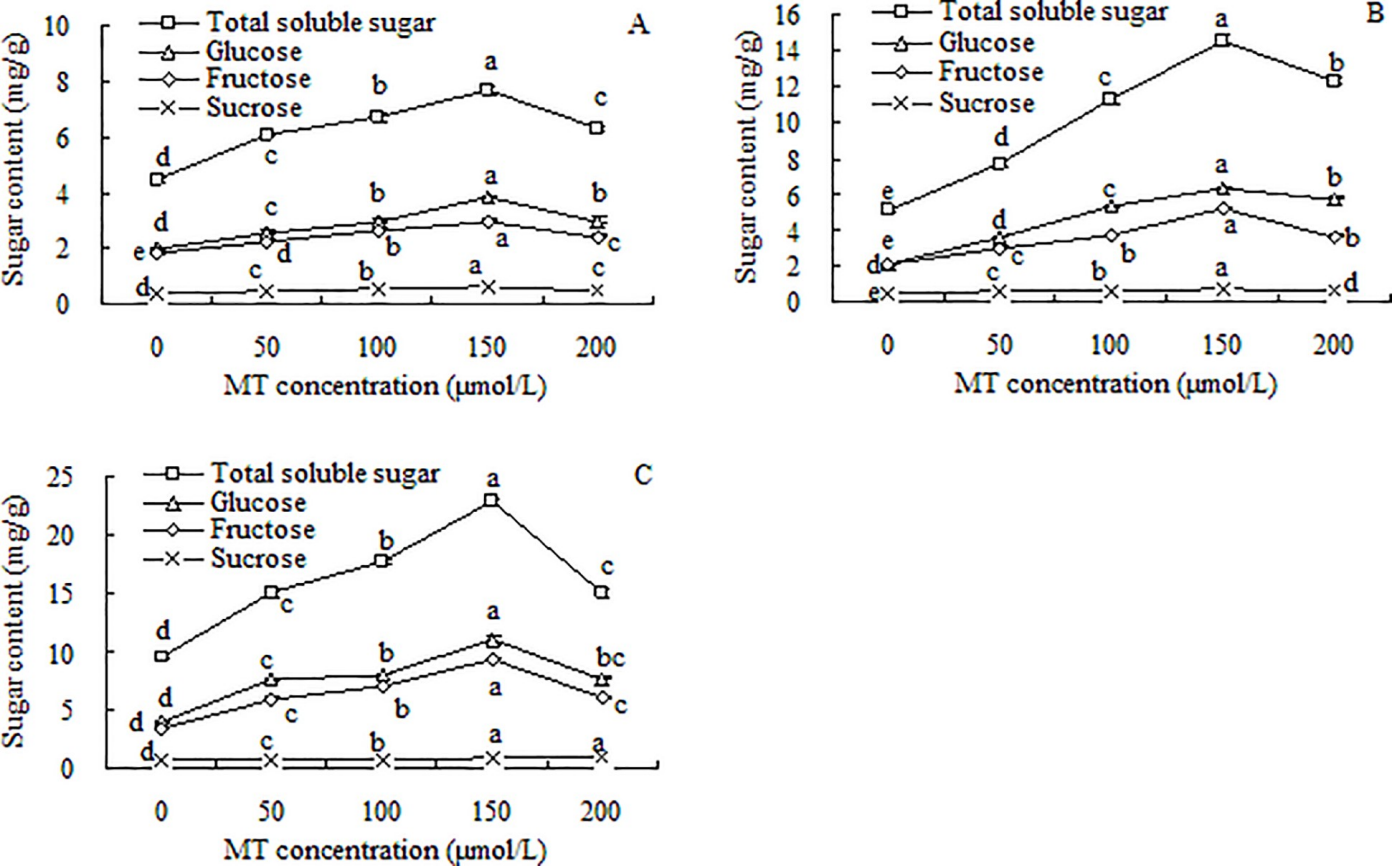
Sugar contents in roots (A), stems (B), and leaves (C) of grape seedlings. Different lowercase letters indicate significant differences based on one-way analysis of variance followed by least significant difference test (*p* ≤ 0.05).

The different types of sugars in roots, stems, and leaves could be ranked, from most to least abundant, as follows: soluble sugars > glucose > fructose > sucrose (Fig 2A, 2B and 2C). The highest contents of sugars were in the leaves, followed by the stems and then the roots. The trends in the contents of total soluble sugars, glucose, fructose, and sucrose were basically the same among the different tissues: as the MT concentration increased, the sugar contents gradually increased up to the MT concentration of 150 μmol/L, then decreased in the 200 μmol/L MT treatment. The highest total soluble sugars contents in the roots, stems, and leaves of grape seedlings were increased by 71.04% (*p* < 0.05), 179.79% (*p* < 0.05), and 140.18% (*p* < 0.05), respectively, compared with the control.

The glucose contents in the roots, stems, and leaves were 3.84, 6.33, and 11.09 mg/g, respectively, accounting for 50.01%, 43.54% and 48.62% of the total soluble sugars content in the corresponding tissues. The fructose contents in roots, stems, and leaves were 2.99, 5.20, and 9.37 mg/g, respectively, accounting for 38.81%, 35.73% and 41.08% of the total soluble sugars content in the corresponding tissues. The sucrose contents in roots, stems, and leaves were 0.601, 0.685, and 0.911 mg/g, respectively, which accounted for only 7.83%, 4.71% and 3.99% of the total soluble sugars contents in those tissues. Although MT promoted the accumulation of sucrose, the sucrose contents were still significantly lower than the glucose and fructose contents. These results showed that the soluble sugars in the grape seedlings were mainly glucose and fructose at a ratio of about 1:1, with a small proportion of sucrose.

### Activities of sucrose-metabolizing related enzymes in grape seedlings

**Fig 3:**
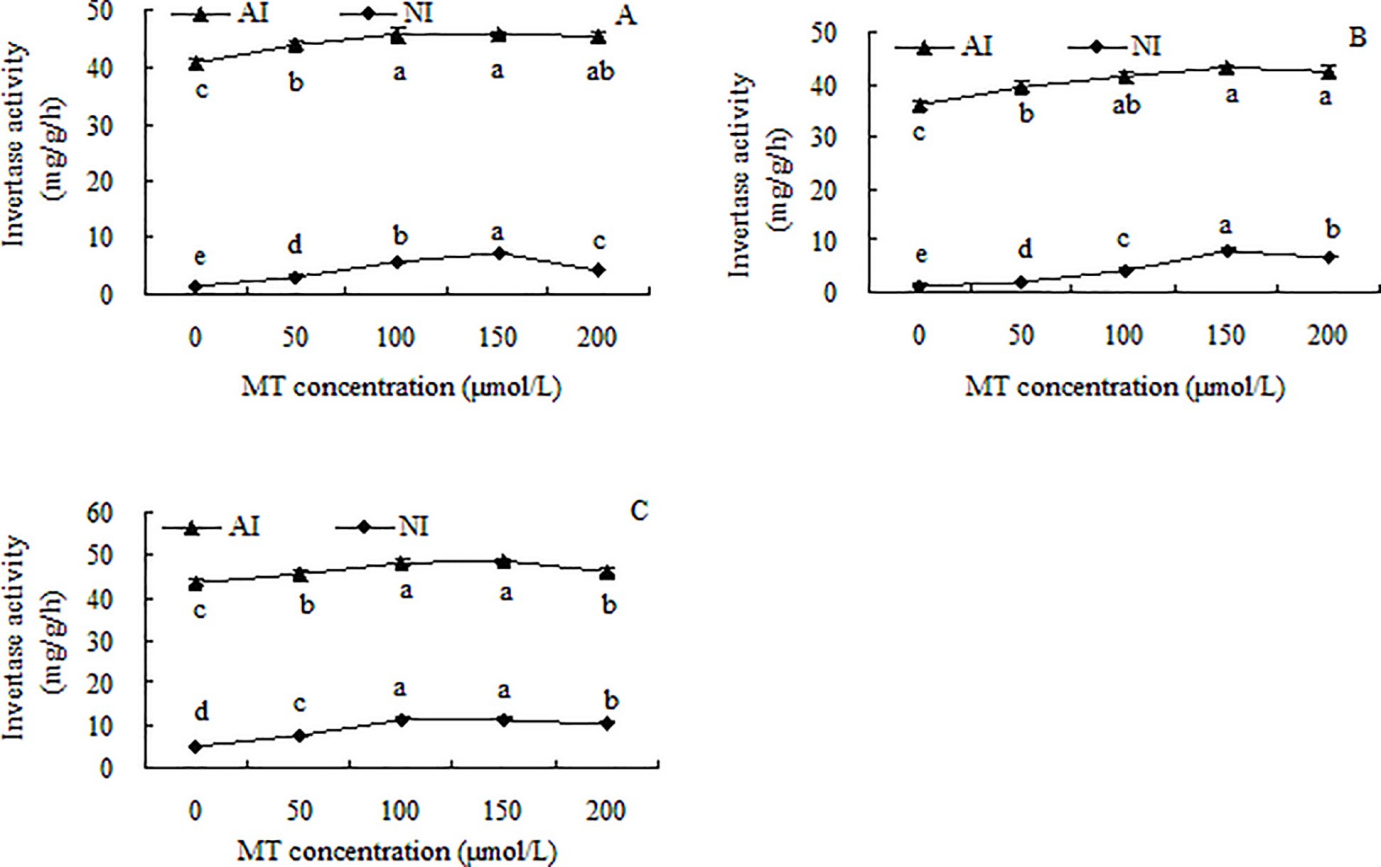
Invertase activities in roots (A), stems (B), and leaves (C) of grape seedlings. Different lowercase letters indicate significant differences based on one-way analysis of variance followed by least significant difference test (*p* ≤ 0.05). AI = acid invertase; NI = neutral invertase.

In the roots, stems, and leaves of grape seedlings, at the same MT concentration, the AI activity was much higher than the NI activity (Fig 3A, 3B and 3C). The trends in AI activities were similar in all tested tissues. When the MT concentration was ≤ 100 μmol/L, the AI activities increased significantly with increasing MT concentration. The differences in AI activities between the 100 and 150 μmol/L MT treatments were not significant, but AI activity was significantly lower in the 200 μmol/L MT treatment. The AI activity was higher in all the MT treatments than in the control. In the 150 μmol/L MT treatment, the AI activity in the roots, stems, and leaves was increased by 12.17% (*p* < 0.05), 19.93% (*p* < 0.05), and 11.22% (*p* < 0.05), respectively, compared with the control. The trends in NI activity were similar to those of AI activity. The NI activity generally increased with increasing MT concentration, peaked, and then decreased. The highest NI activities were in the 150 μmol/L MT treatment, when activities in the roots, stems and leaves were 348.51% (*p* < 0.05), 460.40% (*p* < 0.05), and 118.86% (*p* < 0.05) higher, respectively, than in the control.

**Fig 4:**
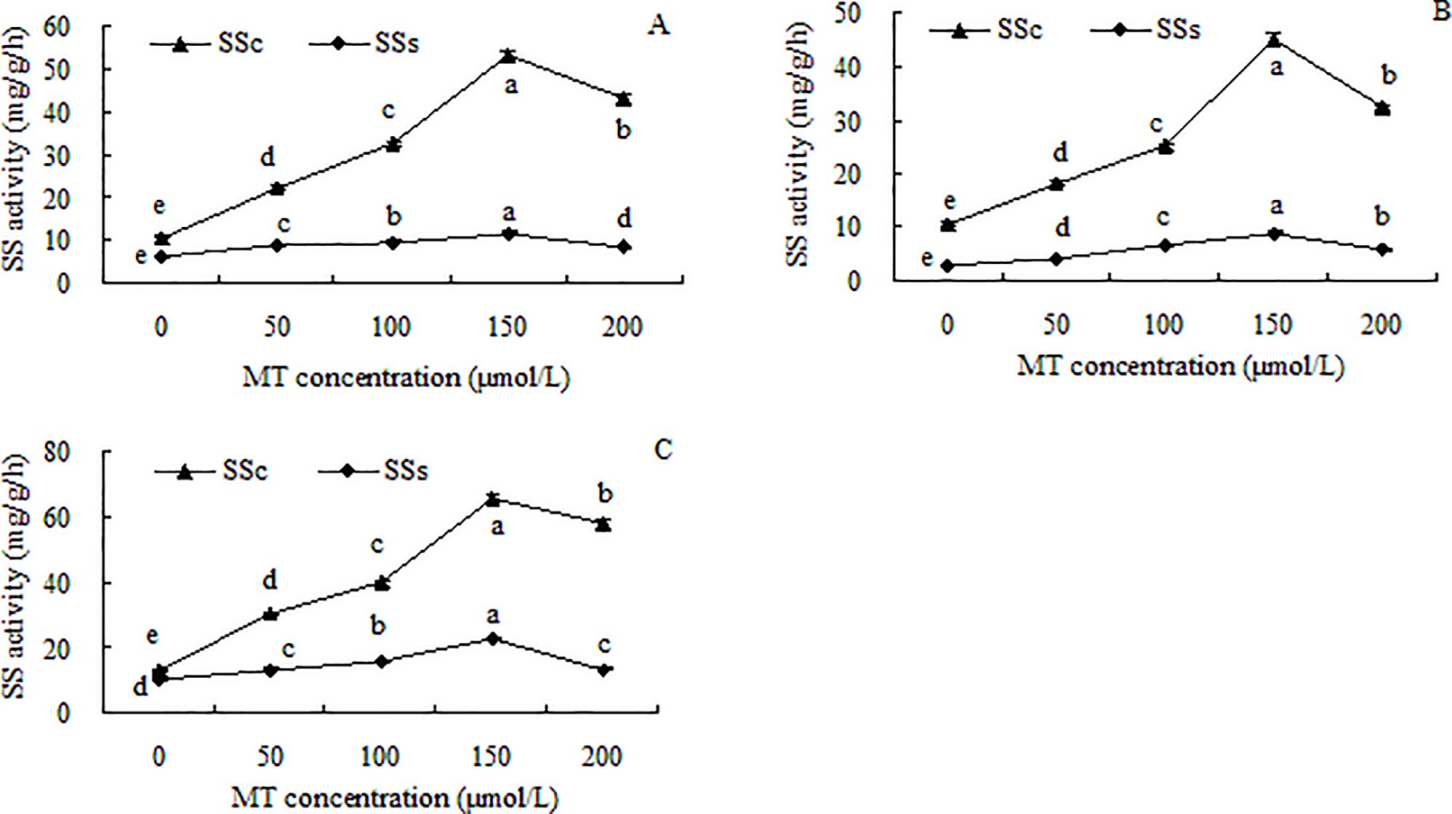
Sucrose synthase (SS) activities in roots (A), stems (B), and leaves (C) of grape seedlings. Different lowercase letters indicate significant differences based on one-way analysis of variance followed by least significant difference test (*p* ≤ 0.05). SSc = sucrose synthase in cleavage direction; SSs = sucrose synthase in synthetic direction.

The trends in SSc and SSs activities in grape seedlings were similar. Both showed increasing activity with increasing MT concentrations, peak activity in the 150 μmol/L treatment, and lower activity in the 200 μmol/L MT treatment (Fig 4A, 4B and 4C). In all the MT treatments, SSc activity was higher than SSs activity, and as the MT concentration increased, the difference between the two gradually increased. At the maximum activities of SSc and SSs, the SSc activities in roots, stems, and leaves were 4.55 times, 5.03 times, and 2.93 times higher, respectively, than SSs activities.

**Fig 5:**
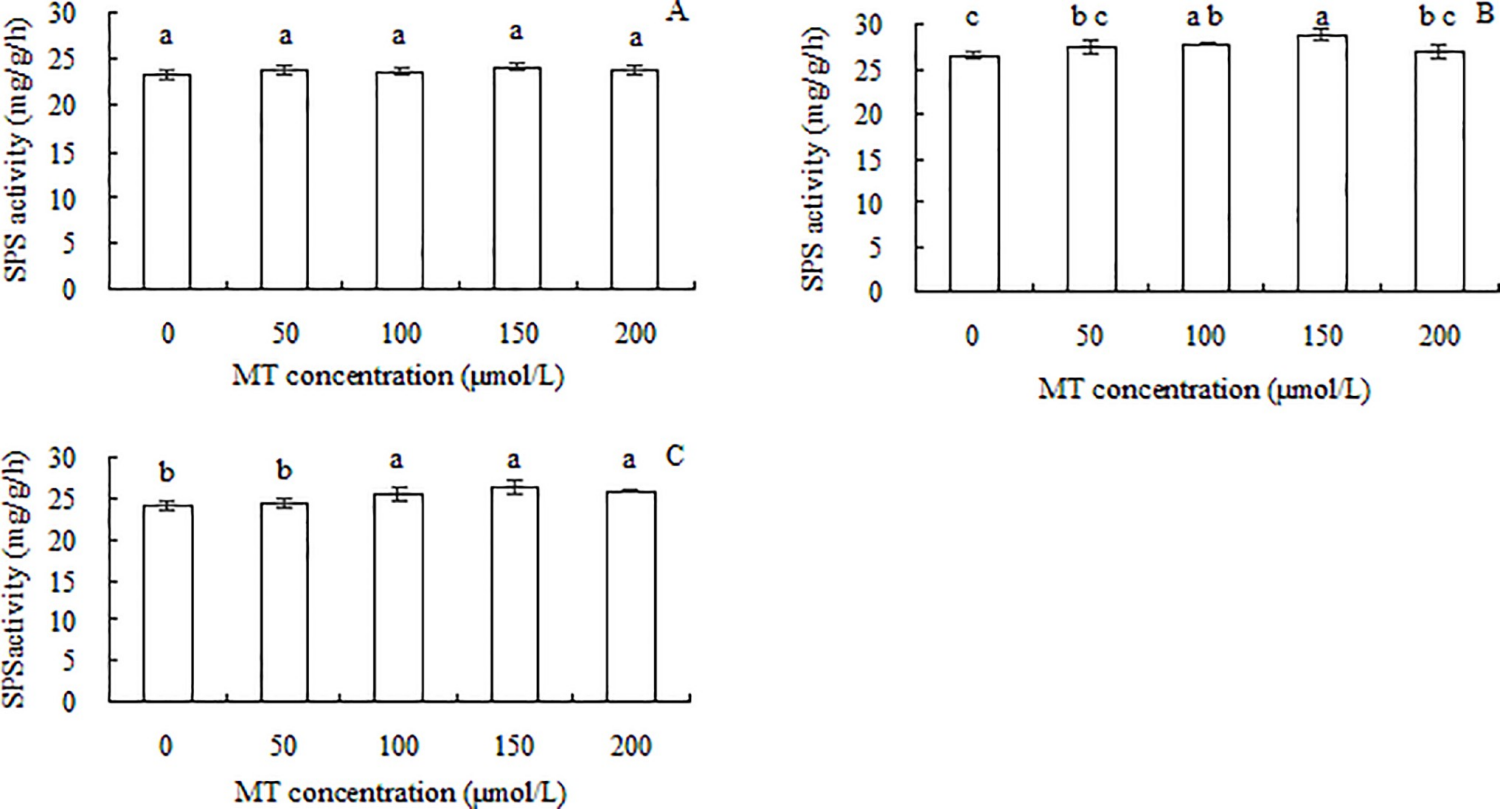
Sucrose phosphate synthase (SPS) activities in roots (A), stems (B), and leaves (C) of grape seedlings. Different lowercase letters indicate significant differences based on one-way analysis of variance followed by least significant difference test (*p* ≤ 0.05).

The maximum values of the ordinates showed that the activities of SPS were not significantly different among roots, stems, and leaves (Fig 5A, 5B and 5C). There was no significant difference in SPS activities in roots among the different MT treatments (Fig 5A). As the concentration of MT increased, the activity of SPS in stems increased slightly, peaked in the 150 μmol/L MT treatment, then decreased significantly in the 200 μmol/L MT treatment (Fig 5B). The SPS activity in the leaves was not significantly different between the 50 μmol/L MT treatment and the control, but was significantly higher in all other MT treatments than in the control. The SPS activity did not differ significantly among the 100, 150, and 200 μmol/L MT treatments (Fig 5C). In conclusion, MT treatments had little effect on SPS activity in grape seedlings.

## Discussion

### MT promotes growth of grape seedlings by regulating IAA content and promoting photosynthetic performance

Root elongation and leaf extension benefit plant growth, and studies have shown that MT can promote plant growth [34–35]. In this experiment, the root biomass of grape seedlings was significantly affected by the MT treatments, with low concentrations of MT having a promoting effect and high concentrations having an inhibitory effect. The effects were very similar to those of IAA [6]. In fact, studies have shown that the function of MT to promote root growth is closely related to changes in IAA content [7]. In previous studies, application of exogenous MT stimulated the root growth of etiolated seedlings of *Brassica juncea*, while the IAA content in the plant was increased by 1.4 times [36]; in tomato seedlings, 50 μmol/L MT showed the best effect to induce root formation by regulating IAA and nitric oxide signalling, and the IAA content in MT-treated plants was double that in the control [37]. In those cases, the promoting effect of MT on plant growth may have been due to increases in the IAA content. However, in other studies, the IAA content in plants decreased after MT treatments [38–39]. This may because exogenous MT induces the synthesis of MT in plants; MT and IAA share a common precursor, tryptophan, and competition for the precursor can lead to a decrease in IAA content [40]. In that case, the effect of MT to promote plants growth may have been due to changes in the abundance of the auxin flux protein AUXIN1/LIKE-AUX1(AUX/LAX), which regulates the development of lateral roots and root hairs, and root orientation. In this way, MT may affect local IAA gradients and interfere with auxin action [41]. Studies have shown that MT has significant effects on the formation of lateral and adventitious roots, but not the main root [42–43]. In the present study, the grape seedlings were derived from cuttings, and the newly grown roots were lateral roots. The MT treatments significantly promoted the formation and growth of lateral roots of grape seedlings, so that the root biomass increased.

The MT treatments also increased the biomass of shoots (stems and leaves). The promoting effects of MT treatments on grape shoot growth may have been via its effects on calcium channels [44]. Another possible reason for the increased shoot biomass in the MT treatments is the higher photosynthetic parameters and photosynthetic pigment contents of grape seedling leaves. Studies have shown that MT can down-regulate the expression of genes encoding chlorophyll-degrading enzymes, thereby delaying the degradation of chlorophyll and leaf senescence [45–47]. In addition, MT can reduce the degradation of carotenoids, increase the Gs of plant leaves, and increase the ability of leaves to absorb CO_2_ [48–49]. Together, these effects of MT may have led to the delayed senescence of grape seedling leaves, allowing them to maintain high photosynthetic performance for a longer period of time, and ultimately leading to increased shoot biomass.

### MT improved resistance of grape seedlings by increasing antioxidant enzyme activity and contents of osmotic adjustment substances

Previous studies have shown that MT plays an important role in the response of plants to drought [50], extreme temperature [51–52], salt [53] and heavy metal stresses [54–55]. Melatonin can protect plants from abiotic stresses because it is a very potent free radical scavenger [56]. Experiments have shown that MT can directly scavenge free radicals such as ·OH, H_2_O_2_, ^1^O_2_, and some of the products of those reactions are also effective free radical scavengers. Thus, MT can scavenge free radicals in plants very effectively [57–58]. As the first line of defence against oxidative stress, MT not only directly scavenges free radicals, but also protects plants by increasing the activities of antioxidant enzyme activities and the contents of osmotic adjustment substances. In previous studies, treatments with 10–500 μmol/L exogenous MT significantly decreased the MDA content while increasing the activities of CAT and POD in *Isatis indigotica* fort. seedlings, which protected the plants from oxidative damage under low temperature stress [59]. Exogenous Treatments with MT also increased the contents of proline, soluble protein, and soluble sugars in leaves, and alleviated low temperature damage to cell membranes by reducing the relative conductivity and MDA content of melon leaves [60]. In our study, MT treatments significantly increased the activities of SOD, POD, and CAT and the soluble protein contents of grape seedling leaves, and decreased the MDA contents and relative conductivity, consistent with the results of previous studies [59–60].

### MT stimulated enzymes related to sucrose metabolism to increase sugar content in grape seedlings

Sugars play multiple roles in the metabolism, growth, and development of plants. They are products of photosynthesis and substrates for respiration, and can enhance the resistance of plants. In higher plants, “source” tissues (mainly mature leaves) synthesise carbohydrates by photosynthesis and then transport them to non-photosynthetic “sink” tissues (e.g. flowers, fruits, seeds, and roots) through the phloem in the form of sucrose. Upon reaching the “sink” tissues, sucrose is hydrolysed to hexose or its derivatives, which are further used in various metabolic and biosynthetic processes [16]. In these processes, sucrose-metabolizing enzymes play a key role. Invertases, including AI and NI, catalyse the hydrolysis of sucrose to glucose and fructose in sucrose metabolism. Therefore, high invertase activity leads to higher glucose and fructose contents [61–62]. Studies have shown that high invertase activity in plants is associated with rapid tissue growth. In rapidly growing tissues, AI activity is always higher than NI activity [63–64]. In this study, the changes in glucose and fructose contents in roots, stems, and leaves of grape seedlings were basically consistent with the changes of invertase activities in those organs, and AI activity was much higher than NI activity, consistent with previous studies [63–64]. Studies have shown that AI is an important enzyme for sugar accumulation in grape berry [60,65]. In the present study, AI activity in roots, stems, and leaves of grape seedlings was increased in all the MT treatments, indicating that AI is also a key enzyme for the growth and development of vegetative organs of grape seedlings.

In addition to sucrose invertase, SS can also hydrolyse sucrose to glucose and fructose, but this process is reversible [18]. In this study, SSc activities were significantly higher than SSs activities in all tissues and in all MT treatments. These results indicate that SS mainly acted to hydrolyse sucrose, consistent with the high contents of glucose and fructose. The sucrose content was also increased by MT treatments. This may be due to SPS activity: that is, MT treatments increased the activity of SPS, a key enzyme for sucrose synthesis, in the stems and leaves of grape seedlings, thus increasing sucrose production in the shoots. Under the action of sucrose-decomposing enzymes (AI, NI, and SSc), most of the sucrose was hydrolysed. This is reflected by the fact that sucrose accounted for only small proportions of total sugars in all treatments.

In general, as the MT concentration increased, the photosynthetic performance of grape leaves increased, resulting in greater sucrose synthesis. Because MT stimulated the activities of enzymes related to sucrose metabolism, most of the sucrose was converted into glucose and fructose, leading to high contents of those sugars and low contents of sucrose.

## Conclusions

In this study, we evaluated the effects of different concentrations of MT on the growth and sucrose metabolism of grape seedlings. The MT treatments resulted in increased biomass, photosynthetic performance, and photosynthetic pigments contents of grape seedlings, compared with the control. The MT treatments also resulted in increased antioxidant enzyme activities and higher concentrations of osmotic adjustment substances. The sugar contents and activities of glucose metabolism-related enzymes were also higher in the MT treatments than in the control. The results of this study indicate that there is mutual influence and a promotional relationship between the growth of grape seedlings and their sugar metabolism. Because MT promotes the growth and development of roots, stems, and leaves of grape seedlings, they are better able to absorb nutrients and compete for resources. The MT treatments promoted photosynthesis in grape seedling leaves, leading to greater sucrose synthesis. The enzymes related to sucrose metabolism were also stimulated by MT, so that most sucrose was hydrolysed into glucose and fructose, which provided materials for growth and development. The resistance of grape seedlings was also increased by MT treatments, because of increased total soluble sugar contents and higher antioxidant enzyme activities. Under these conditions, the optimal MT concentration for treating grape seedlings was 150 μmol/L.
